# ScN/GaN(11̅00): A New Platform for the Epitaxy of Twin-Free Metal–Semiconductor Heterostructures

**DOI:** 10.1021/acs.nanolett.4c00659

**Published:** 2024-05-17

**Authors:** Philipp John, Achim Trampert, Duc Van Dinh, Domenik Spallek, Jonas Lähnemann, Vladimir M. Kaganer, Lutz Geelhaar, Oliver Brandt, Thomas Auzelle

**Affiliations:** Paul-Drude-Institut für Festkörperelektronik, Leibniz-Institut im Forschungsverbund Berlin e.V., Hausvogteiplatz 5-7, 10117 Berlin, Germany

**Keywords:** GaN/ScN heterostructures, core/shell nanowires, twin-free epitaxy, transition metal nitrides, metal−semiconductor heterostructures, uniaxial strain, cubic GaN

## Abstract

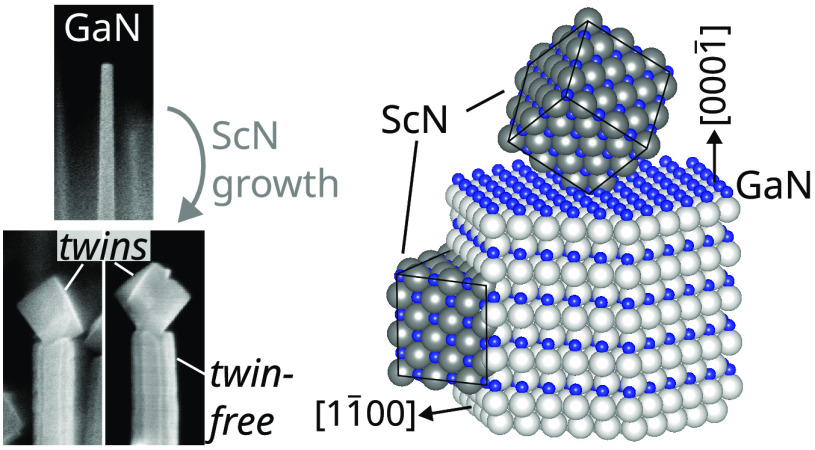

We study the molecular beam epitaxy of rock-salt ScN on the wurtzite GaN(11̅00) surface. To this end, ScN is grown on freestanding GaN(11̅00) substrates and self-assembled GaN nanowires exhibiting (11̅00) sidewalls. On both substrates, ScN crystallizes twin-free thanks to a specific epitaxial relationship, namely ScN(110)[001]∥GaN(11̅00)[0001], providing a congruent, low-symmetry interface. The 13.1% uniaxial lattice mismatch occurring in this orientation mostly relaxes within the first few monolayers of growth by forming a near-coincidence site lattice, where 7 GaN planes coincide with 8 ScN planes, leaving the ScN surface nearly free of extended defects. Overgrowth of the ScN with GaN leads to a kinetic stabilization of the zinc blende phase, that rapidly develops wurtzite inclusions nucleating on {111} nanofacets, commonly observed during zinc blende GaN growth. Our ScN/GaN(11̅00) platform opens a new route for the epitaxy of twin-free metal–semiconductor heterostructures including closely lattice-matched GaN, ScN, HfN, and ZrN compounds.

To enhance the functionalities of conventional group III_*A*_-nitride (III_*A*_-N) devices that are based on the semiconductors AlN, GaN, and InN, combinations with transition metal nitrides (TMNs) like NbN, ScN, ZrN, and TiN are sought.^1−3^ These “new nitrides” bring in distinctive properties such as superconductivity,^2^ ferroelectricity,^4^ catalytic activity,^5^ and plasmonic resonances in the visible spectrum.^6,7^ This opens up new prospects for enhancing the performance of current devices or creating innovative new ones. To fully realize this potential, successful heterostructuring of the TMNs with III_*A*_-N semiconductors will necessitate epitaxial integration with a low density of extended defects. However, while the hexagonal wurtzite phase is the most stable one for III_*A*_-nitrides, the cubic rock-salt structure is the common phase for TMNs. Due to the different crystal symmetry, rotational twins are inevitable for the growth of TMNs on the (0001) plane of hexagonal GaN, SiC, or Al_2_O_3_^8^—the substrates most commonly used for III_*A*_-N heterostructures and devices. Twin domain boundaries locally reduce the crystal symmetry and may thus exhibit dramatically different properties compared to the bulk. In the context of optoelectronic devices, remarkable examples are superconducting twin boundaries in otherwise insulating WO_*x*_ (with *x* ≈ 3) or twin boundaries hosting polarization singularities in otherwise nonpolar perovskite structures.^9^ Concerning TMNs, preliminary observations suggest that twin boundaries in NbN are not superconducting, which will lower the overall critical temperature of the material.^10^

To achieve single-domain TMN layers on hexagonal GaN substrates, we investigate here the epitaxy of ScN on the (11̅00) surface (also referred to as the *M*-plane surface), since it has a lower symmetry than the conventional (0001) surface (also referred to as the *C*-plane surface). (11̅00) facets are of practical relevance as they can be realized on large substrates (≥2 inches) either dislocation-free on the sidewalls of most GaN micro- and nanostructures prepared top-down and bottom-up^11−14^ or with dislocations by heteroepitaxy on γ-LiAlO_2_ substrates.^15,16^ Here, ScN is merely used as a model system because it can be grown lattice-matched to GaN^3,17,18^ and, unlike most transition metals, Sc can be evaporated using an effusion cell. We find that ScN grows free of twins on GaN(11̅00) thanks to a specific epitaxial relationship. We obtain this finding by growing twin-free ScN both on free-standing GaN(11̅00) substrates and self-assembled GaN nanowires. These two types of substrates facilitate the use of different analytical techniques, yielding consistent results. The uniaxial strain occurring in ScN/GaN(11̅00) is relaxed via a near-coincidence site lattice, making the ScN layers suitable for regrowth of twin-free cubic nitrides.

ScN is first grown by plasma-assisted molecular beam epitaxy (PAMBE) on self-assembled GaN nanowires (NWs) under N-rich conditions to impede the formation of N-vacancies.^19,20^ Representative secondary electron micrographs of a GaN NW before and after the growth of a 25 nm ScN shell are displayed in Figure 1a,b, respectively. ScN deposited on the (0001̅) NW top facet forms a cubic crystallite, whereas ScN grown on the (11̅00) NW sidewalls forms a relatively smooth layer. The ScN shell and cube exhibit different crystallographic orientations, which are related to their distinct epitaxial relationships with the underlying GaN facet. As schematized in Figure 1e, the ScN cube follows the same epitaxial relationship as commonly found for ScN on GaN(0001) substrates, namely ScN(111)[11̅0]∥GaN(0001̅ )[112̅0].^17,18,20^ Twinning is observed between cubes located on different NWs or even within the same NW (the latter not shown here). In contrast, a very different epitaxial relationship is found for the ScN shell on the GaN NW sidewalls. Figure 1c shows a high-resolution transmission electron microscopy (HRTEM) image of a 10 nm thick ScN shell taken with the zone axis parallel to the [112̅0]_GaN_ direction. No twin domains are found after analyzing ≈10 NWs. Comparison with the simulation shown in the inset of Figure 1c reveals the orientation-relationship ScN(110)[001]∥GaN(11̅00)[0001]. This finding is confirmed by plan-view HRTEM along the [0001]_GaN_ zone axis. Figure 1d reveals a smooth ScN shell nucleating on well-defined GaN{11̅00} facets. Since the GaN/ScN orientation-relationship holds on every facet of the NW core, the ScN nucleating on adjacent {11̅00}_GaN_ facets is separated by high-angle boundaries, leading eventually to the formation of vertical grooves as observed in Figure 1b. The orientation-relationship reported here has already been observed between ScN and AlN as a result of thermal decomposition of (Sc,Al)N thin films,^21^ but it is not systematic for all rock-salt compounds grown on wurtzite (11̅00) planes (e.g., MgO on ZnO^22^). Interestingly, the same out-of-plane orientation, but rotated 90° in-plane, was reported for some TMNs grown on Al_2_O_3_(11̅00).^23,24^

**Figure 1:**
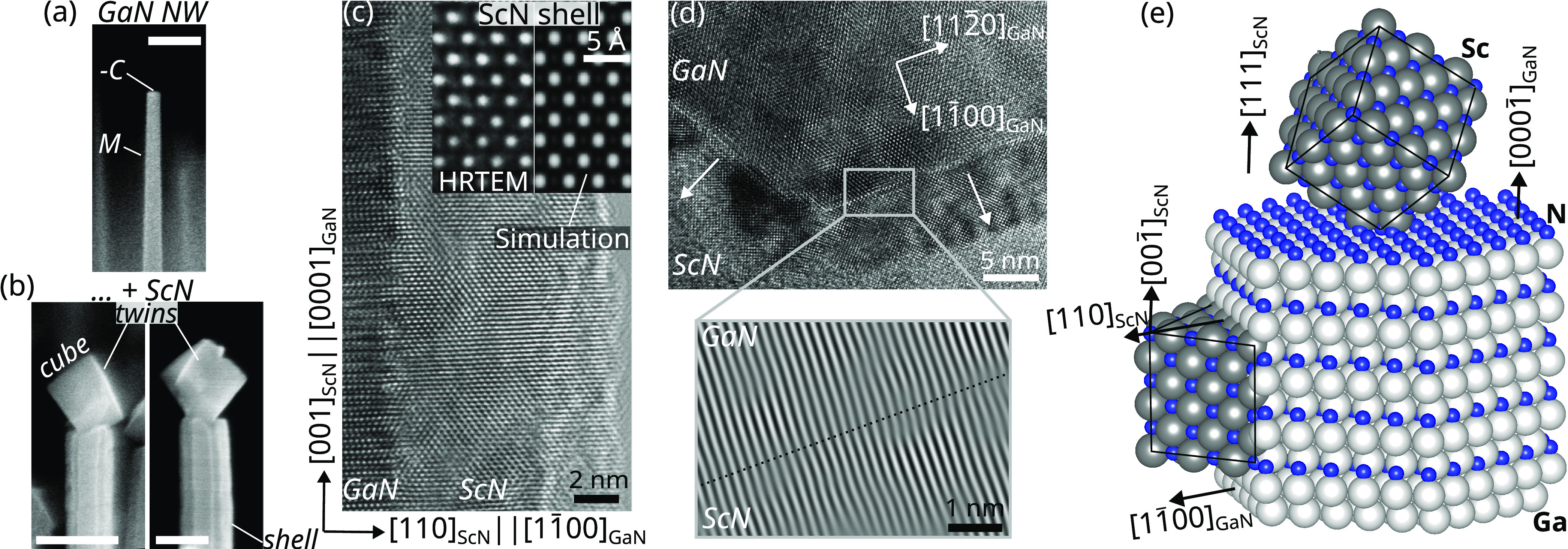
Secondary electron micrographs of GaN NWs (a) before and (b) after overgrowth with a 25 nm thick ScN shell. The scale bars indicate 200 nm. (c) HRTEM of the interface between the GaN core and a 10 nm thick ScN shell with [112̅0]_GaN_ zone axis. The inset compares a magnified HRTEM image with a simulation using the epitaxial relationship described in the main text. (d) HRTEM along the NW axis, i.e., with [0001]_GaN_ zone axis. White arrows within the ScN shell indicate ScN[110] directions. An inverse Fourier-filtered section from the GaN/ScN interface, highlighting GaN(112̅0) and ScN(11̅0) planes, is shown in the bottom panel. (e) Schematic representation of the local GaN/ScN epitaxial relationships at the NW tip and sidewall.

To confirm the epitaxial relationship and the twin-free nature of ScN on a large scale, a 40 nm thick film is grown on a freestanding GaN(11̅00) substrate. On such a reference surface, illustrated in the atomic force topograph of Figure 2a, the ScN orientation is monitored *in situ* by reflection high-energy electron diffraction (RHEED). The patterns taken along the [11̅0]_ScN_ and [001]_ScN_ azimuths (Figure 2c,d, respectively) perfectly match the ones simulated using the epitaxial relationship found for the NW shell and shown by black dots. The presence of 45° chevrons in the pattern along the [001]_ScN_ azimuth indicates that the layer is terminated by {100} facets.^25^ In N-rich conditions, such {100} facets are stoichiometric^26,27^ and are typically the ones of lowest energy in rock-salt compounds.^28^ The nanofaceting of the ScN layer is confirmed by the atomic force topograph shown in Figure 2b, evidencing an anisotropic surface morphology consisting of periodic stripes, which are oriented along the [001]_ScN_ direction. We attribute this anisotropic surface structure to an anisotropy in the adatom diffusion barriers along the ScN [001] and [11̅0] directions.

**Figure 2:**
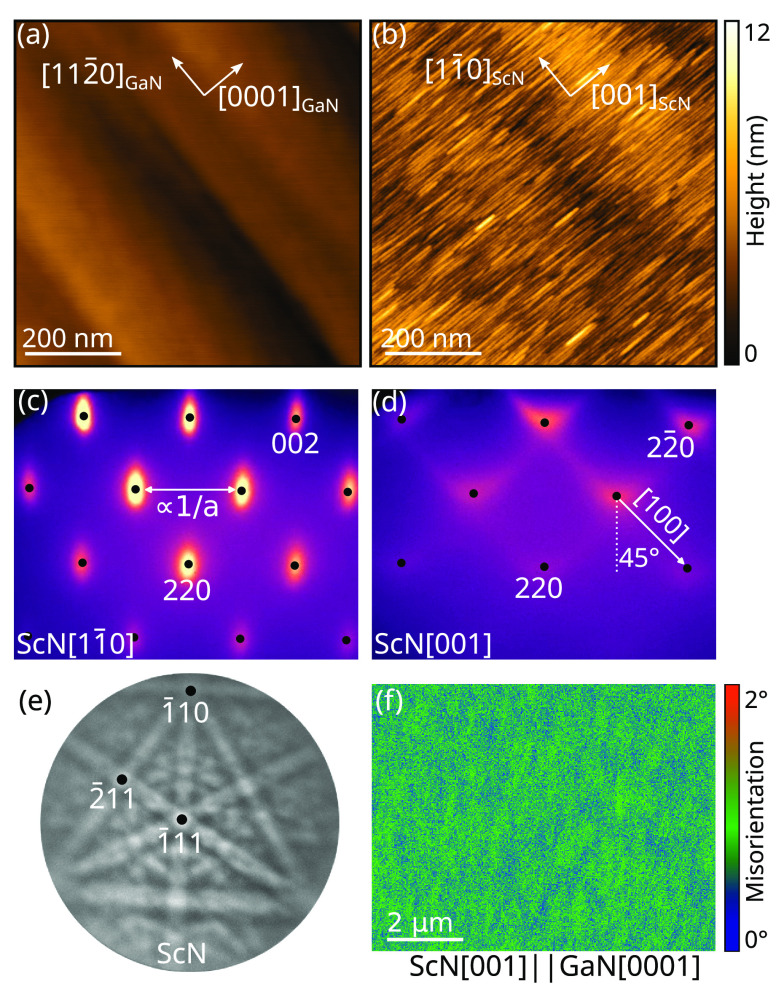
Atomic force topographs of (a) a freestanding GaN(11̅00) substrate and (b) a 40 nm thick ScN layer. (c,d) RHEED patterns and superimposed diffraction simulations (black dots) along the ScN azimuths indicated in the bottom left corner. (e) EBSD pseudo-Kikuchi pattern and (f) in-plane orientation map of the ScN layer, where variations in color show the local deviation from the nominal ScN orientation indicated below.

To confirm the absence of small inclusions with a different epitaxial relationship, the ScN film is probed at the μm scale with electron backscatter diffraction (EBSD). The pseudo-Kikuchi pattern exemplified in Figure 2e is used to locally identify the crystal orientation.^29^ It is composed of sharp bands, enabling fast and reliable indexing when mapping large areas. Figure 2f shows the resulting EBSD orientation map, visualizing the in-plane orientation of the ScN film with a spatial and angular resolution of 20 nm and 1°, respectively. Indeed, the film is single crystalline with a tilt distribution close to the resolution limit, thus revealing the absence of any additional domains.

Careful analysis of both the NW shells and the thin films indicates the absence of twin domains. This can be understood by looking at the atomic configuration of the ScN/GaN interface, schematized in Figure 3a. For the experimentally determined epitaxial relationship, GaN and ScN are lattice-matched along the [112̅0]_GaN_ direction but display a 13.1% mismatch along the [0001]_GaN_ direction. No other favorable atomic configuration can be found by rotating or mirroring the ScN lattice in the interfacial plane, and the mirror symmetry of GaN(11̅00) matches that of ScN(110), which are the two necessary conditions to achieve single-domain ScN layers.^8^ One competing epitaxial relationship, however, would be that observed for ScN deposited on GaN(0001) (e.g., as seen for MgO on ZnO NWs^22^). Indeed, for ScN on GaN(11̅00), such a configuration would lead to a very low epitaxial strain ( %), but the mismatch between the *AB*_GaN_ and *ABC*_ScN_ plane stacking sequence would most likely result in a much higher density of dangling bonds, as schematized in the Supporting Information. Therefore, the most favorable interfacial atomic configuration appears here to be the one that minimizes the number of dangling bonds, but with significant epitaxial strain.

**Figure 3:**
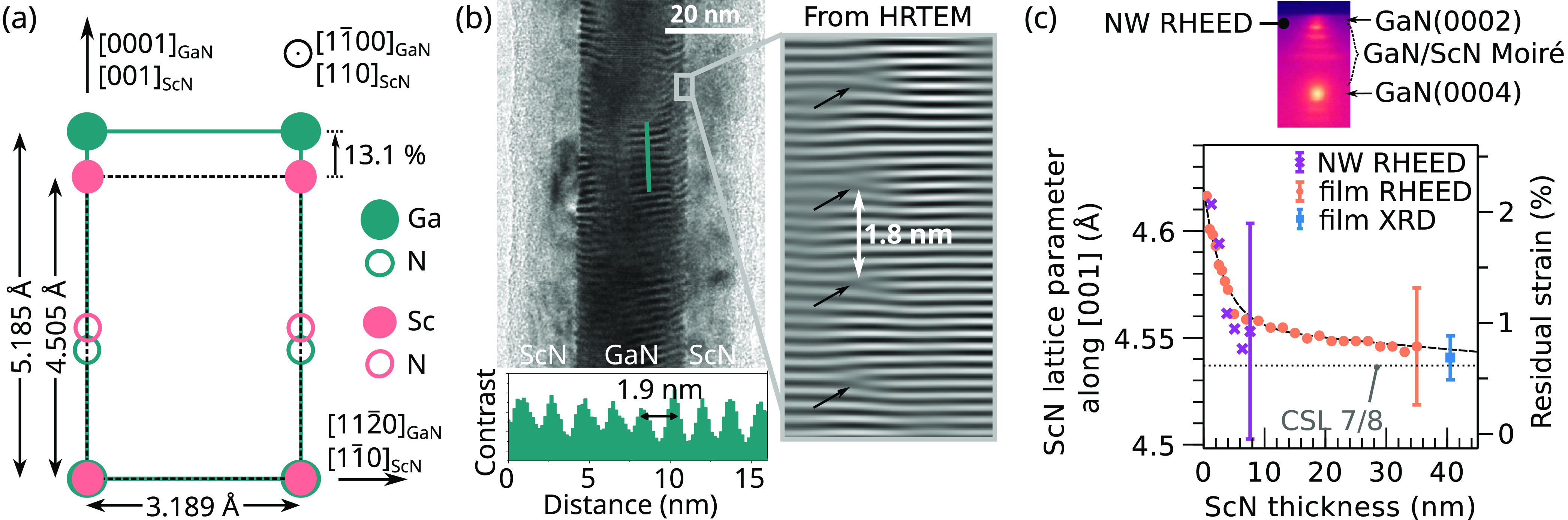
(a) Schematic atomic arrangement of two-dimensional ScN(110) and GaN(11̅00) unit meshes. (b) TEM of a GaN/ScN core/shell NW along the [112̅0]_GaN_ zone axis revealing moiré patterns. An intensity profile taken along the green line is shown in the bottom panel, and an inverse Fourier-filtered section from a HRTEM image of the interface, highlighting GaN(0002) and ScN(002) planes, is shown in the right panel. Misfit dislocations are indicated by black arrows. (c) ScN lattice parameter measured in films and NWs with RHEED and XRD along the [001]_ScN_ direction, as a function of layer thickness. The error bars represent the errors for all data points of the respective measurements. The top panel exemplifies a section of the RHEED pattern during GaN/ScN core/shell NW growth along the [11̅00]_GaN_ azimuth.

The critical thickness for plastic relaxation of the huge uniaxial lattice mismatch (13.1%) imposed by the GaN substrate on the ScN layer is less than one monolayer (ML). We thus expect the ScN layer to quickly approach its strain-free lattice parameter taken as 4.505 Å.^30^ Consistently, moiré patterns are observed during the TEM observations of GaN/ScN core/shell NWs (Figure 3b), resulting from the superposition of two lattices with different lattice parameters.^31^ The moiré period along the NW axis ([001]_ScN_ direction) is 1.9 ± 0.1 nm, which corresponds to a residual strain of 1.3 ± 0.7% for the 12 nm thick ScN shell.

Inverse Fourier filtering of an HRTEM image of the ScN/GaN interface is used to highlight GaN(0002) and ScN(002) planes at the GaN/ScN interface. It evidences a regular array of geometric misfit dislocations with a period of  nm, as displayed in the right panel in Figure 3b, *d* indicating the respective interplanar lattice distance. In contrast, misfit dislocations are absent along the [112̅0]_GaN_ direction (see bottom panel in Figure 1d), where the ScN lattice matches that of GaN. These geometric dislocations are thus of pure edge (90°) character and their period of 7/8 lattice planes can be explained in the framework of a near-coincidence site lattice (n-CSL) model,^32^ for which a residual strain state of  % is expected. In contrast to grain boundaries in metals,^33^ such periodic arrays of pure edge dislocations are typically observed in highly mismatched heterostructures^34−37^ and do not necessarily lead to a large density of threading dislocations.^37^ Here, no obvious sign for threading dislocations nor stacking faults could be seen within the  NWs examined by TEM.

Next, we closely monitor the kinetics of the strain relaxation using RHEED.^38^ In the thin film case, the ScN lattice parameter in the [001]_ScN_ direction is directly retrieved from its reciprocal space lattice measured along the [11̅0]_ScN_ azimuth (cf. Figure 2c) and taking *c*_GaN_ = 5.185 Å for the initial GaN substrate.^39^ In the NW case, satellites around the GaN Bragg peaks occur, as displayed in the top panel of Figure 3c. These satellites are the result of the successive transmission of the electron beam through the NW core and shell and can be seen as the Fourier transform of the core/shell GaN/ScN moiré pattern with p* = a* + c*, where p*, a*, and c* are the reciprocal lattice vectors of the moiré pattern, ScN shell and GaN core, respectively, averaged over  NWs.

The ScN lattice parameter and residual strain along its [001] axis, extracted from the RHEED analysis of the film and the NWs, are displayed in Figure 3c as a function of the ScN thickness. For both layers and NWs, plastic relaxation is observed already at the onset of ScN growth. The strain of the first ScN monolayer is already reduced to 2.0 ± 0.5%. Such immediate strain reduction could be explained by the formation of an 8/9 n-CSL that releases a lattice mismatch of 11.1%. For further ScN growth of up to 40 nm in thickness, the strain decreases and tends to saturate at a minimum value of  %, coinciding with the expected value for the n-CSL of 7/8 lattice planes for GaN and ScN. This value is also confirmed by X-ray diffractometry (XRD) performed on the 40 nm-thick ScN film.

Further or complete strain relaxation implies an interruption of the CSL periodicity, which would require additional energetic activation, explaining the apparent saturation of the residual strain at a value of 0.7% for thin ScN layers. As a result, the top surface of the 40 nm-thick ScN layer grown on GaN(11̅00) exhibits a uniaxial strain of  %, but essentially no extended defects like twin-domain boundaries, stacking faults, or threading dislocations. This is particularly favorable for regrowth of cubic transition metal nitride heterostructures on GaN with high structural perfection. Note that extended defects, such as threading dislocations, may be introduced as a result of further ScN plastic relaxation at later growth stages.^32^

An interesting question is whether the epitaxial relationship found for ScN grown on GaN(11̅00) holds also when GaN is regrown on ScN. To clarify this point, GaN is first deposited on GaN/ScN core/shell NWs and representative TEM micrographs of the resulting GaN/ScN/GaN core/shell structure are shown in Figure 4. The 3 nm thick GaN shell is rougher than the underlying ScN(110) surface and contains both wurtzite (α-GaN) and zinc blende (β-GaN) phases (Figure 4b). The cubic β-GaN domains share the same orientation as the underlying ScN, namely β-GaN(110)[001]∥ScN(110)[001], resulting in a perfectly commensurate interface (Figure 4c). The low energy of the interface is certainly a key factor in explaining the kinetic stabilization of the otherwise thermodynamically unstable zinc blende phase. Surprisingly, the wurtzite crystal phase does not follow the epitaxial relationship found for ScN deposited on GaN(11̅00). Instead, α-GaN inclusions nucleate on stacking faults (SFs) preferentially formed on {111}_β-GaN_ facets, as commonly observed in rough β-GaN films.^40,41^ The epitaxial relationship at the wurtzite–zinc blende interface is then given by α-GaN(0001)[112̅0]∥β-GaN(111)[11̅0]. This translates into the effective orientation of α-GaN(11̅03)[112̅0]∥ScN(110)[11̅0] with the underlying ScN. The same orientation has been observed for α-GaN inclusions occurring during the growth of β-GaN on MgO(110)^42^ and GaAs(110).^43,44^ From geometric considerations taking the angle between the respective lattice planes, a 3.3° tilt between the α-GaN[11̅03] and ScN[110] directions is expected in the absence of strain.

**Figure 4:**
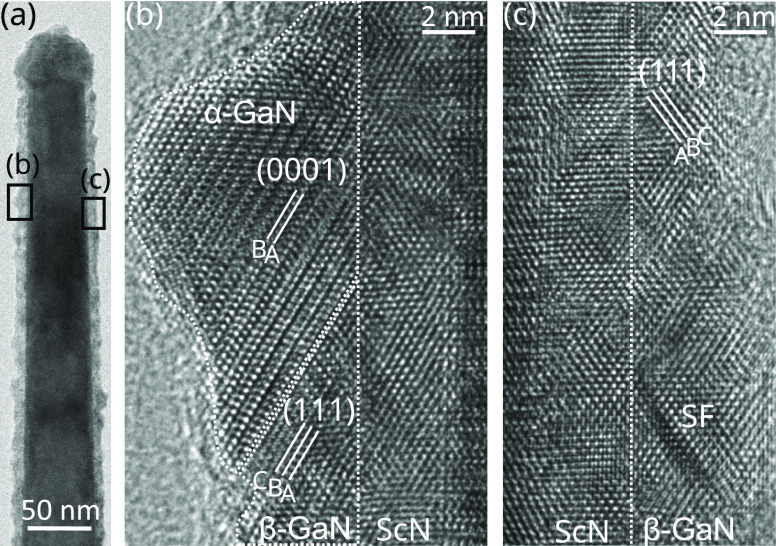
TEM of a GaN/ScN core/shell NW overgrown by GaN, along the [112̅0]_GaN_ zone axis of the NW core. (a) Overview showing a rough NW shell. Magnified images in (b) and (c) reveal the presence of mixed α-GaN/β-GaN and pure β-GaN domains, respectively.

Continued GaN growth leads to an increase in the size of the wurtzite inclusions, which eventually cover the entire surface. This is evidenced by growing 25 and 250 nm-thick GaN layers on ScN/GaN(11̅00) films. EBSD patterns and phase maps of the two layers are displayed in Figure 5a,b, respectively. The thinner GaN layer exhibits mostly the zinc blende phase, whereas the thicker one shows mostly the wurtzite phase. In spite of the metal-rich growth conditions, the atomic force topograph acquired on the thicker GaN layer and shown in Figure 5c reveals a faceted surface, most of them being attributed to wurtzite {0001} and {11̅00} facets, from which we conclude that α-GaN{11̅03} facets are unlikely to be stabilized. Two different in-plane α-GaN orientations are found, resulting from the nucleation on both (111) and (111̅) facets of the initial β-GaN layer. A symmetric 2θ/ω XRD scan of the 250 nm GaN film is displayed in Figure 5d. It evidences the presence of both β-GaN(220) and α-GaN{11̅03} reflections, the latter being tilted by 2.5° compared to the α-GaN[11̅00] substrate direction (see inset of Figure 5d). The reduced tilt compared to the theoretical expectation of 3.3° is attributed to residual strain in the ScN and top GaN layers. The asymmetry of the β-GaN(220) peak results from the superposition with the underlying ScN(220) reflection being nearly lattice-matched.

**Figure 5:**
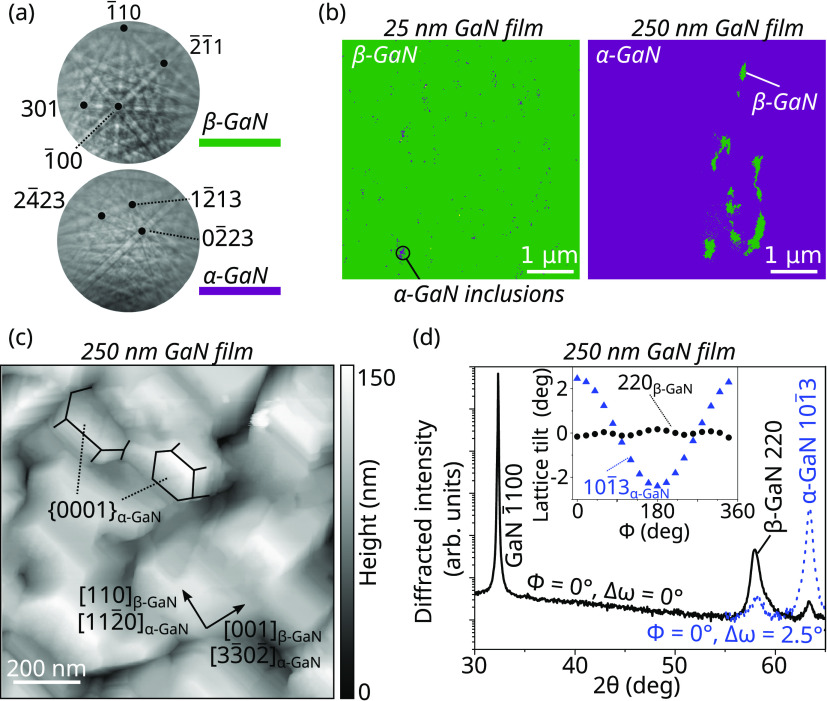
EBSD patterns (a) and phase maps (b) acquired on the 25 and 250 nm thick GaN layers grown on ScN(110)/GaN(11̅00) heterostructures. (c) Atomic force topograph of the 250 nm thick GaN layer. (d) XRD 2θ/ω scan of the same layer, revealing the presence of α-GaN and β-GaN, tilted relative to each other. The inset shows the lattice tilt of α-GaN and β-GaN as a function of azimuth ϕ, evidencing  of α-GaN(101̅3) relative to the substrate.

Our results indicate that thin III_*A*_-nitrides like GaN, AlN, and InN can be kinetically stabilized in their metastable β phase on ScN(110), thus enabling new metal–semiconductor heterostructures. However, the formation of wurtzite inclusions, a common problem during the growth of cubic III_*A*_-nitrides, should be prohibited by using specific growth conditions as previously documented for growth on Si(001),^45^ GaAs(001),^46^ and 3*C*-SiC.^47^ For instance, stabilizing a smooth β-GaN(110) surface using surfactants^48^ could be decisive to prevent the formation of {111}_β-GaN_ facets where α-GaN inclusions nucleate.^41^ On the other hand, new twin-free TMN heterostructures containing e.g. ScN, HfN, and ZrN are suitable to be grown on our ScN/GaN(11̅00) platform, since TMNs are thermodynamically stable in the cubic rock-salt phase.

In conclusion, we have studied the growth of GaN/ScN heterostructures on the low-symmetry GaN(11̅00) surface, both on NW sidewalls and on free-standing substrates. Twin-free ScN(110) layers are obtained using this approach due to a specific epitaxial relationship for which the interface symmetries match that of bulk ScN(110). The uniaxial epitaxial strain imposed by the substrate along the GaN[0001] direction plastically relaxes within the first MLs, leaving a residual strain of  % for ScN layers up to a thickness of 40 nm. Subsequent deposition of GaN on ScN/GaN(11̅00) results in a kinetic stabilization of β-GaN that rapidly transforms into a twinned α-GaN layer nucleating on {111}_β-GaN_ facets. As a result, the epitaxial relationship for α-GaN on ScN drastically differs from that of ScN on α-GaN. Importantly, our ScN/GaN(11̅00) platform provides a new route for the epitaxy of twin-free heterostructures combining semiconductors and metals like β-GaN, ScN, HfN, and ZrN, which are all nearly lattice-matched.^3^

## Methods

ScN shells between 3 and 100 nm thickness were grown in N-rich conditions by PAMBE on GaN NWs at 840 °C and with a substrate rotation speed of 2–3 rpm. Active N was provided by a plasma cell with an atomic flux of 1.5 × 10^15^ s^–1^ cm^–2^, and Sc by a high-temperature effusion cell with an atomic flux of (1–2) × 10^14^ s^–1^cm^–2^. The N and Sc fluxes were calibrated by determining the growth rate of GaN and ScN layers grown in N-limited and Sc-limited regimes, respectively. The temperature was calibrated as described in ref (49). GaN NWs were grown via self-assembly on sputtered TiN films decorated with Si seeds, as further described in ref (50). This approach allows fabricating ensembles with a NW density below 10^9^ cm^–2^, which is useful to prevent shadowing between neighboring NWs during the shell growth.

ScN layers with a thickness between 25 and 40 nm were grown on freestanding 5 × 10 mm^2^ GaN(11̅00) substrates at 740 °C with atomic fluxes similar to those used for the NWs. A 100 nm thick GaN layer was deposited prior to the ScN to create smooth atomic steps.

GaN overgrowth on ScN was done at 740 °C, on NW shells with a III/V ratio of 0.4 and on ScN films in slightly Ga-rich conditions.

The orientation and lattice parameter of the growing films were monitored *in situ* by RHEED with an acceleration voltage of 20 kV and *ex situ* by XRD using a PANalytical X’Pert PRO MRD diffractometer. EBSD was carried out in a Zeiss Ultra-55 scanning electron microscope operated at 15 kV and equipped with an EDAX Hikari Super EBSD camera to identify different crystal phases and orientations. Last, the microstructure of dispersed NWs and of plan-view specimens (preparation described in ref (51)) was characterized by TEM in a Jeol 2100F field emission microscope operated at 200 kV and equipped with a Gatan Ultra Scan 4000 charge coupled device.

Diffraction patterns and HRTEM images were simulated using the JEMS software package, the latter by applying a multislice approach.

## Supporting Information Available

Schematic atomic arrangement of ScN on GaN(0001) and GaN(11̅00) surfaces (PDF)
