# Supplementary material for: ScN/GaN(11̅00): A New Platform for the Epitaxy of Twin-Free Metal–Semiconductor Heterostructures
Source: Nano Lett. 2024 May 17;24(21):6233–9. doi: 10.1021/acs.nanolett.4c00659 (PMC11140757; doi:10.1021/acs.nanolett.4c00659)
Supplement: Supplementary file 1 — nl4c00659_si_001.pdf [file nl4c00659_si_001.pdf]

Supporting Information: ScN/GaN(1$\bar{1}$00): a new platform for the epitaxy of twin-free metal-semiconductor heterostructures

Philipp John,* Achim Trampert, Duc Van Dinh, Domenik Spallek, Jonas Lähnemann, Vladimir M. Kaganer, Lutz Geelhaar, Oliver Brandt, and Thomas Auzelle

Paul-Drude-Institut für Festkörperelektronik, Leibniz-Institut im Forschungsverbund Berlin e.V., Hausvogteiplatz 5-7, 10117 Berlin

E-mail: john@pdi-berlin.de

The common orientation of epitaxial ScN on *C*-plane GaN, as also observed in this work for ScN grown on GaN nanowire top facets, is described by ScN(111)[1$\bar{1}$0]||GaN(0001)[11$\bar{2}$0]. The corresponding GaN(0001) and ScN(111) unit meshes, schematized in plan-view in Figure S1(a), exhibit the same atomic arrangement and quasi identical lattice parameters. The superposition of these planes thus results in a commensurate interface with negligible epitaxial strain. Nevertheless, twinning is inevitable due to the different GaN(0001) and ScN(111) rotational symmetries (6-fold and 3-fold, respectively), which are defined by the stacking of the subsequent monolayers not visible in the unit meshes depicted in Figure S1(a).

If the same orientation was kept for ScN grown on the GaN(1$\bar{1}$00) surface, the corresponding orientation-relationship would be described by ScN(11$\bar{2}$)[1$\bar{1}$0]||GaN(1$\bar{1}$00)[11$\bar{2}$0], leading to a hypothetical interface as schematized in Figure S1(b) in cross-sectional view. Although the inter-planar lattice distances along the GaN[0001] and ScN[111] directions match, the mismatch in AB_{GaN} and ABC_{ScN} stacking sequence leads to different atomic positions at the interface. Indeed, the dimensions of the corresponding GaN(1$\bar{1}$00) and ScN(11$\bar{2}$) plan-view unit meshes shown in Figure S1(c) show an enormous mismatch ($> 50\,\%$) along the GaN[0001] and ScN[111] directions. Epitaxy in this orientation would require a strong distortion of the interfacial bonds, likely leading to a high density of dangling bonds. Since the superposition of these planes does not lead to a reasonable interface, a ScN(11$\bar{2}$) orientation on GaN(1$\bar{1}$00) is unlikely.

Instead, the epitaxial relationship experimentally observed in this work is given by ScN(110)[001]||GaN(1$\bar{1}$00)[0001], as explained in the main manuscript and reproduced in Figure S1(d). Although this orientation involves a 13.1 % uniaxial lattice mismatch, the fast strain relaxation via the coincidence site lattice including a periodic array of misfit dislocations (see main manuscript) seems to lead to an energetically more favorable interface.

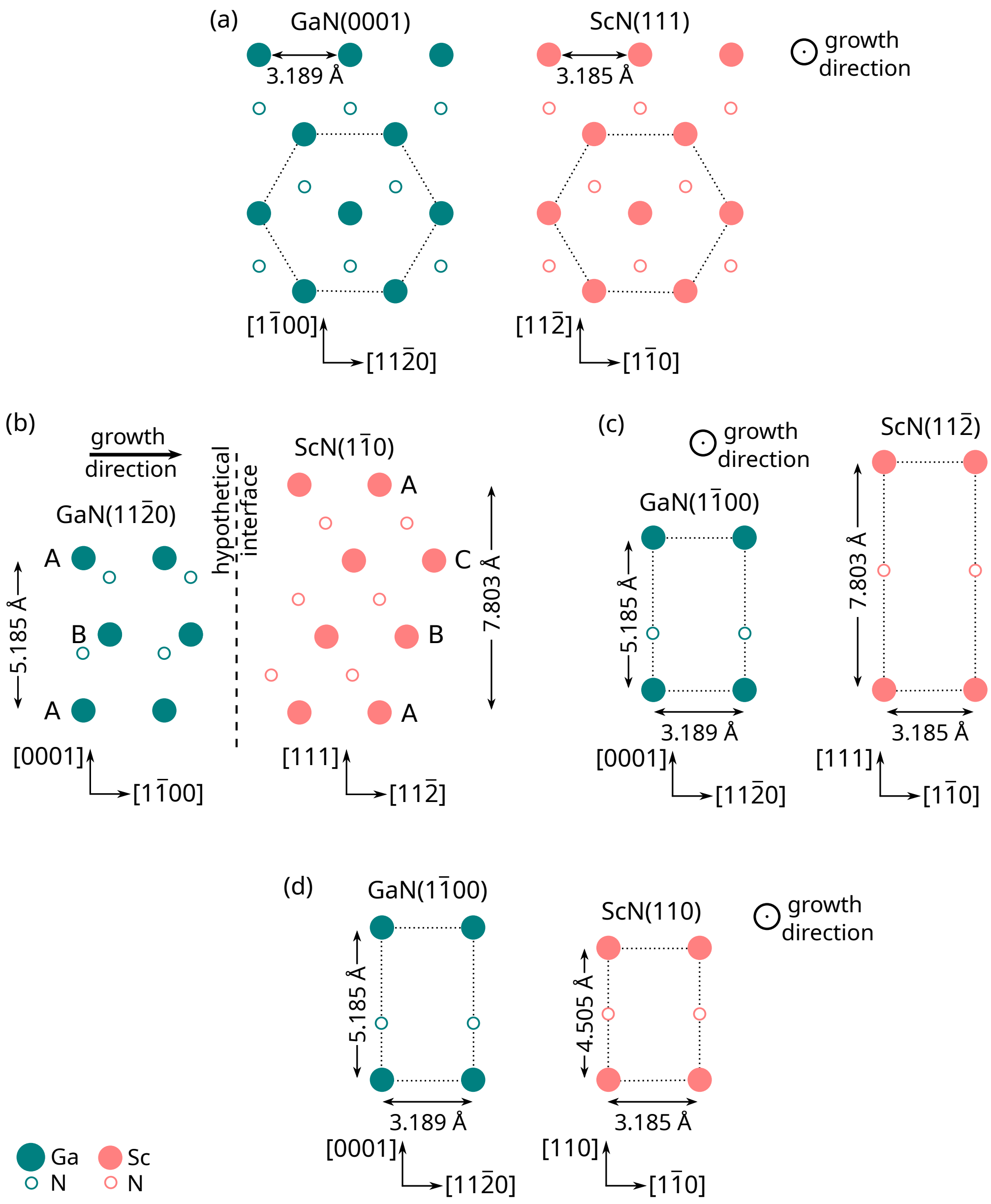

Figure S1: (a) Schematic two-dimensional GaN(0001) and ScN(111) unit meshes. (b) Schematic atomic arrangement of a hypothetical GaN(1$\bar{1}$00)/ScN(11$\bar{2}$) interface (in cross-sectional view), where (c) shows the corresponding GaN(1$\bar{1}$00) and ScN(11$\bar{2}$) plan-view unit meshes, which would need to be superimposed to keep the epitaxial relationship that is found for ScN on GaN(0001). (d) Two-dimensional GaN(1$\bar{1}$00) and ScN(110) unit meshes, showing the actual epitaxial relationship obtained in this work.
